# Improvement of Skin Penetration, Antipollutant Activity and Skin Hydration of 7,3′,4′-Trihydroxyisoflavone Cyclodextrin Inclusion Complex

**DOI:** 10.3390/pharmaceutics11080399

**Published:** 2019-08-08

**Authors:** Pao Hsien Huang, Stephen Chu Sung Hu, Feng Lin Yen, Chih Hua Tseng

**Affiliations:** 1 School of Pharmacy, College of Pharmacy, Kaohsiung Medical University, Kaohsiung City 807, Taiwan; 2 Department of Dermatology, College of Medicine, Kaohsiung Medical University, Kaohsiung City 807, Taiwan; 3 Department of Dermatology, Kaohsiung Medical University Hospital, Kaohsiung City 807, Taiwan; 4 Drug Development and Value Creation Research Center, Kaohsiung Medical University, Kaohsiung City 807, Taiwan; 5 Department of Fragrance and Cosmetic Science, College of Pharmacy, Kaohsiung Medical University, Kaohsiung City 807, Taiwan; 6 Institute of Biomedical Sciences, National Sun Yat-Sen University, Kaohsiung City 804, Taiwan; 7 Department of Medical Research, Kaohsiung Medical University Hospital, Kaohsiung City 807, Taiwan; 8 Department of Pharmacy, Kaohsiung Municipal Ta-Tung Hospital, Kaohsiung City 801, Taiwan

**Keywords:** 7,3′,4′-trihydroxyisoflavone, 2-Hydroxypropyl-β-cyclodextrin, antioxidant, solubility, skin penetration, skin hydration

## Abstract

As is known, many antioxidants from plant extracts have been used as additives in skincare products to prevent skin damage following overexposure to environmental pollutants. 7,3′,4′-trihydroxyisoflavone (734THIF), an isoflavone compound, possesses various biological activities, including antioxidant, antityrosinase, photodamage protection, and anticancer effects. Unfortunately, 734THIF has poor water solubility, which limits its skin penetration and absorption, and subsequently influences its biological activity. The aim of the present study was to investigate the mechanisms for the improvement in water solubility and skin penetration of 2-hydroxypropyl-β-cyclodextrin (HPBCD) inclusion complex with 734THIF (5-7HP). We also determined its photostability, antipollutant activity in HaCaT keratinocytes, and moisturizing effect in human subjects. Our results showed that 734THIF was embedded into the lipophilic inner cavity of HPBCD and its water solubility and skin penetration were thereby improved through amorphous transformation, surface area enhancement, and hydrogen bonding formation between 734THIF and HPBCD. In addition, 5-7HP inhibited PM-induced ROS generation and then downregulated ROS-mediated COX-2 and MMP9 production and AQP-3 consumption by inhibiting the phosphorylation of MAPKs. Consequently, we suggest that 5-7HP is a safe and photostable topical ingredient to enhance the skin penetration of 734THIF and skin hydration, and therefore 5-7HP may be used as an antipollutant additive in skin care products.

## 1. Introduction

Air pollution has gradually increased recently and is an important issue that the World Health Organization (WHO) is concerned about. According to an investigation in 2018 by WHO, air pollution causes seven million deaths every year and 91% of people in the world live in areas that exceed air pollution standards [1]. Air quality is evaluated by the level of carbon dioxide (CO_2_), sulfur oxides (SO_x_), nitrogen oxides (NO_x_), carbon monoxide (CO), and particulate matters (PMs). PMs, the major pollutant, are composed of sulphate, nitrates, ammonia, sodium chloride, black carbon, mineral dust, polycyclic aromatic hydrocarbons (PAHs), and metals in the air. Several articles have indicated that PMs cause human body damage including cardiovascular diseases [2], respiratory symptoms [3], and skin aging, which includes wrinkle formation and pigmentation [4,5]. 

The skin is the first line of defense against external substances such as PMs. When the skin is exposed to “exogenous-aging PM factors” for a long time, it is easy to induce reactive oxygen species (ROS) and inflammatory proteins (e.g., COX-2) and downregulate moisture proteins (e.g., filaggrin) [6] to cause skin aging and damage. ROS mediate mitogen-activated protein kinase (MAPK) signaling, transcription factor nuclear factor (NF)-κB and activating protein-1 (AP-1), leading to induction of proinflammatory cytokines and the matrix metalloproteinases (MMPs) family. The MMPs family is a main culprit for skin wrinkling production by causing collagen downregulation [4]. In addition, skin aging is also associated with loss of skin moisture. Aquaporin 3 (AQP-3), an important protein which mediates water content by the transport of water and glycerol, is the most abundantly expressed aquaporin in keratinocytes [7]. Reduced levels of AQP-3 result in impairments in skin hydration, elasticity, glycerol content in skin and is also found in psoriasis patients [8]; however, there is no report regarding whether exogenous aging PMs factors would cause AQP-3 expression to affect skin moisture. Hence, this study was performed.

Recently, many plant extracts and their antioxidants have been used in skincare products for protecting skin damage from environmental pollutant, such as carotenoids and polyphenols [9]. 7,3′,4′-trihydroxyisoflavone (734THIF) is one group of polyphenols and has been shown to possess great benefits to the skin, such as antioxidant [10], moisturizing protein increment [11], and UVB-induced skin cancer inhibition [12]. However, the ability of 734THIF to prevent PM-induced skin aging and its underlying mechanisms is not clear. In addition, according to the solubility definition of United States Pharmacopeia (USP), solubility of 734THIF (<20 μg/mL) belongs to a practically insoluble class of substances that restrict skin penetration and bioavailability. Several studies have used cyclodextrin complexes to overcome the poor water solubility and stability of active compounds, for example, curcumin [13], boswellic acid [14] carvedilol [15], and lansoprazole [16]. 2-Hydroxypropyl-β-cyclodextrin (HPBCD) is a common non-toxic cyclic oligosaccharides carrier that is included in the pharmaceutical ingredient list of the Food and Drug Administration (FDA) [17]. HPBCD has hydrophilic external and hydrophobic internal surfaces both in a cone shape possessing the location to insert hydrophobic drugs and thereby improve physicochemical properties to enhance drug solubility and stability [18,19].

Consequently, the aim of the present study was to investigate the water and phase solubilities, skin penetration, photostability and physicochemical properties of 734THIF and inclusion complex of 734THIF and HPBCD (7HP) by high-performance liquid chromatography (HPLC), powder X-ray diffraction (PXRD), Fourier transformation infrared spectroscopy (FTIR), scanning electron microscopy (SEM), and ^1^H nuclear magnetic resonance (NMR). This study also determines whether 7HP inhibits the mechanism of PM-induced skin aging in the keratinocyte model and 7HP as a cosmetic ingredient to improve effective moisture through a clinical trial.

## 2. Materials and Methods

### 2.1. Materials

734THIF was synthesized by Associate Professor Chih-Hua Tseng (Kaohsiung Medical University School of Pharmacy, Kaohsiung, Taiwan). Hydroxypropyl-beta-cyclodextrin (HPβCD) was purchased from Zibo Qianhui (Shandong, China). HaCaT cell was obtained by Istituto Zooprofilattico Sperimentale della Lombardia e dell’Emilia Romagna (Brescia, Italy). Dulbecco’s modified Eagle’s medium (DMEM) was purchased from Himedia Laboratories (Mumbai, India). Fetal Bovine Serum (FBS) was purchased from Thermo Fisher Scientific (Waltham, MA, USA). Pen-Strep-Ampho.B solution (Penicillin, Streptomycin, amphotericin B) was purchased from Biological Industries (PSA; Connecticut, NE, USA). PM (Standard Reference Material^®^ 1649b) was purchased from the National Institute of Standards and Technology (Gaithersburg, MD, USA) Acetonitrile, dimethyl sulfoxide (DMSO), and methanol were purchased from Aencore Chemical (Surrey Hills, Australia). 3-(4,5-cimethylthiazol-2-yl)-2,5-diphenyl tetrazolium bromide (MTT) was purchased from MDBio (Taipei, Taiwan). Phosphotungstic acid and 2,2-diphenyl-1-picrylhydrazyl (DPPH) were purchased from Sigma (St Louis, MO, USA). Potassium dihydrogen phosphate (KH2PO4) was purchased from Ferak (Berlin, Germany). Primary antibodies were purchased from Abcam (MMP-9 (ab76003), Cambridge, UK), Cell Signaling Technology (COX-2 (12282) and p-JNK (9255), Danvers, MA, USA), Santa Cruze Biotechnology (GAPDH (sc-32233), Dallas, TX, USA), Merck Millipore (p-p38 (09-272), p-erk1/2 (05-797R), Burlington, MA, USA), and Arigo biolaboratories (AQP-3 (ARG10648), Hsinchu, Taiwan).

### 2.2. Methods

#### 2.2.1. Preparation of 734THIF Cyclodextrin Complex (7HP)

734THIF cyclodextrin complex was prepared by a simple co-evaporation method [20]. The molar ratios of HPBCD and 734THIF were 0.1, 0.5, 1.25, 2.5. 3.75, 5, and 10 respectively, with the HPBCD and 734THIF being dissolved in 45 mL of distilled water and in 5 mL of ethanol respectively. Then, the 734THIF ethanol solution was dropped slowly into HPBCD aqueous solution with magnetic stirring at 1200 rpm for 1 h at 25 °C to form a 734THIF cyclodextrin inclusion complex (7HP) solution. Finally, the 7HP solution was placed into a freeze-drying machine to obtain 7HP powder. All different ratios of 734HP were performed in triplicate.

#### 2.2.2. High Performance Liquid Chromatography (HPLC) System

The HPLC system (Hitachi ELITE LaChrom; Hitachi, Tokyo, Japan) consists of an L-2130 pump, L-2200 autosampler and L-2420 ultraviolet (UV)–vis detector. 734THIF concentration was analyzed by Mightysil RP-18 GP (250 × 4.6 mm, 5 μm) column and in a pH 2.8 mobile phase consisting of acetonitrile and 10mM KH_2_PO_4_ (35:65). The flow rate, wavelength of the UV detector, and injection volume were set at 1.0 mL/min, 262 nm, and 20 μL respectively. The calibration curve of 734THIF was linear (r ≥ 0.998) within the range 0.005 to 50 μg/mL. 

#### 2.2.3. Yield and Water Solubility

For yield, different ratios of 7HP were dissolved in methanol. 734THIF concentrations of all samples were determined by HPLC, performed in triplicate, and calculated as follow: Yield (%) = 734THIF weight (mg)/theoretical amount of 734THIF (mg) × 100%.

For water solubility evaluation, different ratios of 7HP were dissolved in water and then shaken for 1 h by Vortex-Gene 2 (Scientific Industries, Bohemia, NY, USA). All samples were filtered with 0.45 μm syringe filter (13 mm Acrodisc^®^ syringe filters with GHP membrane; Pall Corporation, NY, USA) before analysis by HPLC.

#### 2.2.4. Morphology

The surface morphology of 734THIF and different ratios of 7HP powder were observed by scanning electron microscopy (SEM, Hitachi S4700; Hitachi, Tokyo, Japan) and set at 15 kV. All samples were sputter-coated with a thin gold layer by Hitachi E-1045 ion sputter coater (Hitachi, Tokyo, Japan) before analysis.

#### 2.2.5. Powder X-ray Diffraction (XRD)

Crystalline 734THIF and different ratios of 7HP were evaluated by XRD (Siemens D5000; Siemens, Munich, Germany) with Cu-Kα radiation at 40 kV and 80 mA. The scanning angle (2θ) was set from 2° to 50° with the scanning rate at 1°/min.

#### 2.2.6. Fourier-Transform Infrared Spectrometer (FTIR)

FTIR spectra of 734THIF, different ratios of 7HP and HPBCD were obtained from the ALPHA II FTIR spectrometer (Bruker, Billerica, MA, USA), and the scan ranged from 4000 to 400 cm^−1^ with the rate at 2 cm^−1^. About 2–3 mg of each sample were ground evenly with potassium bromide by a mortar and compressed into a thin tablet for analysis. 

#### 2.2.7. Phase Solubility Studies

Phase solubility studies followed the method of Higuchi and Connors [21] and was modified as according to a previous study [22]. An excess of 734THIF was added to 0.1–10 mM HPBCD water solution, then the mixture solutions were stirred at 1000 rpm for 24 h. All samples were filtered with a 0.45 μm syringe filter before analysis by HPLC. The stability constants (Ks) were calculated from the slope of 734THIF solubility in the HPBCD water solution (Slope1) and the intrinsic solubility of 734THIF in water (S0).

Equation (1) is as follows:

Ks = Slope1 × S0 (1 − Slope1)(1)

#### 2.2.8. ^1^H-Nuclear Magnetic Resonance (^1^H-NMR)

HPβCD, 734THIF, and 5-7HP were dissolved in 0.4 mL of DMSO-*d*_6_ and then analyzed by Varian Mercury Plus AS400 ^1^H-NMR System (Oxford Instrument, Abingdon, UK). 

#### 2.2.9. Photostability Assay

Photostability assay was performed by the method of Andonova et al. with some modifications [23]. Solutions of 5-7HP containing 1 mg 734THIF and 1 mL water were exposed to outdoor daylight for 0, 12, 24, 48, 72, 96, 120, 144, and 168 h. In addition, the powder form of 5-7HP containing 1 mg 734THIF was exposed for 0 and 168 h. After light exposure, the samples were filtered with 0.45 μm syringe filter and analyzed by HPLC. 

#### 2.2.10. In Vitro Skin Penetration

Skin penetration assay was modified from the COLIPA guideline for percutaneous absorption/penetration protocol [24]. Skin from the flank region of pigs was purchased from a local market and used for in vitro skin penetration experiments. Before performing the experiments, each skin sample was checked to ensure skin intactness, such as the absence of wounds, ulcers, and abscesses, cut into appropriate sizes (about 2 × 2 cm^2^) and then placed on 10 mm Franz Diffusion Cells (Diffusion surface 0.785 cm^2^ Jacketed, 3.5 mL receptor chambers volume). The system was maintained at 32 °C and stirred at 1000 rpm. The donor chambers were loaded into 200 μL of contented 1 mg/mL 734THIF of 5-7HP water solution and 734THIF water suspension, and treated for 1, 2, 4 and 8 h. Subsequently, the skin was tape-stripped 15 times using 3 M adhesive tape to remove the stratum corneum, and then the skin was placed on a 90 °C heater and cut with a scalpel to separate the epidermis and dermis [25]. All samples were put into methanol and extracted for one hour by ultrasonic cleaner (Branson 5510). Experiments were performed five times and analyzed by HPLC. 

#### 2.2.11. DPPH Scavenging Ability

The DPPH scavenging ability assay was performed according to a previous study [26]. 734THIF in DMSO (734D) and distilled water (734H) and 734HP were reacted with 0.2 M DPPH and then incubated in a dark place for 30 min. The absorbance (A) of reaction solution was determined by a microplate spectrophotometer at 517 nm. The scavenging ability of DPPH was calculated as follows:

Scavenging effect (%) = (A_control_ − A_test_)/A_control_ × 100%

The sample concentration of 50% scavenging activity (SC50) was also evaluated.

#### 2.2.12. Cell Viability Assay

1.5 × 10^4^ HaCaT cells were seeded in 96-well plates per well and cultured in DMEM supplemented with 10% FBS and 1% PSA at 37 °C in 5% CO_2_. After 24 h of incubation, cells were treated with test samples (without FBS) for 24 and 48 h. After that, 150 μL of 0.5 mg/mL MTT solution was added and incubated for 2.5 h at 37 °C for conversion to purple formazan. Finally, the purple formazan of samples was dissolved in DMSO and the absorbance of the plate was evaluated at 550 nm by a microplate spectrophotometer (μQuant; BioTek, Winooski, VT, USA). Experiments were performed in triplicate.

#### 2.2.13. Reactive Oxygen Species (ROS) Assay

For the present study, 1.5 × 10^4^ HaCaT cells were seeded in 96-well plates and incubated at 37  °C for 24 h. Subsequently, cells were pre-treated with samples (HPBCD, 734THIF, and 7HP) for 6 h before reaction with 20 μM dichlorodihydrofluorescein diacetate (DCFH-DA; Sigma, Tokyo, Japan) solution, for detection of intracellular ROS. After 30 min, cells were incubated with 50 μg/cm^2^ PM for an hour, washed twice with PBS, and soaked in PBS for detecting relative fluorescence intensity at excitation and emission wavelengths of 485 nm and 528 nm by fluorescent plate reader (BioTek, Winooski, VT, USA).

#### 2.2.14. Evaluation of PM-Induced Protein Expression in Keratinocytes by Western Blot 

The PM-induced HaCaT cells model was modified from a previous study [6]. PM suspension was sonicated for 5 min. 1 × 10^6^ HaCaT cells were seeded in six-well plates for 24 h and then HPBCD, 734THIF, and 5-7HP solution were added for 24 h before treatment with PM (50 μg/cm^2^) suspension for different periods of time. All drugs were dissolved in DMEM and shaken for 10 min before being filtered. After that, cells were lysed with lysis buffer and centrifuged at 12,000 rpm (Centrifuge 5430 R; Eppendorf, Hamburg, Germany) for 5 min to obtain proteins. Concentrations of proteins were determined with the bicinchoninic acid protein assay kit (BCA, 23225; Thermo Fisher Scientific, Waltham, MA, USA) and proteins samples were separated by SDS–PAGE before transferal to PVDF membrane (Merck Millipore). Subsequently, the membrane was blocked for 1 h, washed with Tris-buffered saline with Tween-20, and incubated with the corresponding primary antibodies including COX-2 (1:1000), MMP-9 (1:1000), GAPDH (1:1000), p-ERK (1:1000), p-p38 (1:1000), p-JNK (1:1000), and AQP-3 (1:1000) at 4 °C overnight. Membranes were then incubated with HRP-conjugated secondary antibody for 1 h at room temperature and reacted with enhanced chemiluminescence reagents before detection by ChemiDoc XRS (Bio Rad, Hercules, CA, USA).

### 2.3. Clinical Trial

#### 2.3.1. Study Subjects

The study recruited 21 volunteers with skin dryness that were more than 20 years old. They were randomized into three groups: Negative control (without isoflavone), test group A (low dose containing 0.3% 734THIF) and test group B (high dose containing 1% 734THIF). Each group had seven volunteers.

#### 2.3.2. Study Design

This study was an 8-week, randomized, single-blind, and parallel clinical trial. Inclusion and exclusion criteria are included in the following Table 1 [27]:

Test lotions contained euxyl^®^ PE9010 (phenoxyethanol/ethylhexylglycerin; Schülke, Norderstedt, Germany), Sepigel 305 (Sodium polyacrylate/C12-16 isoparaffin/Laureth-7; Magicare International Ltd., Tainan, Taiwan) and different concentrations of 5-7HP (0.3 and 1% 734THIF) (Table 2). Volunteers did not use any skin moisturizing product at the beginning of the study and were instructed to use these lotions (2 mg/cm^2^) twice daily on their xerosis area after cleaning for 8 consecutive weeks. After 0, 1, 2, 4 and 8 weeks, subjects underwent skin water content measurement by the non-invasive instrument Cutometer MPA 580 with a skin hydration probe (Corneometer^®^ CM 825, Courage + Khazaka Electronic GmbH, Cologne, Germany). Volunteers were acclimatized to the testing center’s controlled conditions (temperature at 25 ± 2 °C and air humidity at 45–55%) for 15 min before the skin water content measurements.

#### 2.3.3. Ethics

This clinical trial study was performed in accordance with the guidelines of FDA Good Clinical Practice (GCP) and approved by Kaohsiung Medical University Chung-Ho Memorial Hospital Institutional Review Board (Number: KMUHIRB-F-(I)-20160014). All volunteers understood and gave informed consent for participation in the trial. 

### 2.4. Statistical Analysis

For statistical analysis, Microsoft Excel 2010 software (Microsoft Office, Microsoft Corporation, Redmond, WA, USA) and SPSS software version 19 (SPSS Inc., Chicago, IL, USA) were used. All data were expressed as mean ± SD, the significant difference was analyzed using one-way ANOVA with Tukey’s test of variance, and *p*-value < 0.05 was considered to be statistically significant.

## 3. Results

### 3.1. The Optimal Ratio of 7HP Through Complex Formation and Drug Solubility

**Figure 1:**
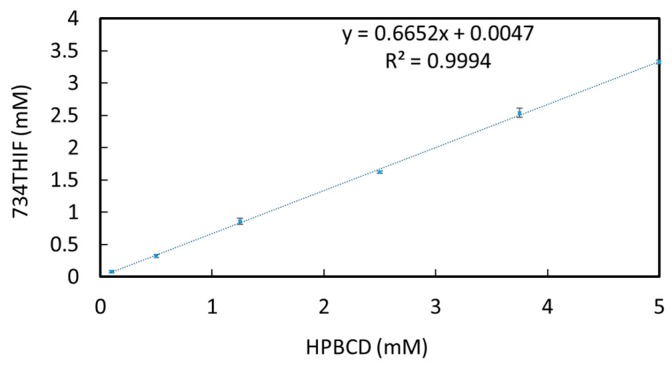
Phase solubility diagram of 734THIF-HPBCD system.

Higuchi and Connors showed that good inclusion complex formation is the major factor for the improvement of drug solubility [28]. Phase solubility analysis is one way to classify the effect of inclusion complexes on substrate solubility. There are major classifications of complex (drug and excipient) phase solubility to evaluate the efficiency of solubility improvement, such as laniary increment (Type A) and without improvement (Type B). As seen in Figure 1, the phase solubility diagram of 734THIF-HPBCD system at 0.1 to 5 mM HPBCD showed a concentration-dependent relationship with HPBCD in solubility enhancement, and the complex phase solubility was Type A. These results indicate that HPBCD is a good excipient to improve the solubility of 734THIF.

After the phase solubility study, the water solubility of 7HPs was measured to determine the optimal ratio of 734HP, and the results are shown in Table 3. Water solubility of 734THIF was 4.00 ± 0.90 μg/mL and increased as the molarity of HPBCD increased. 734THIF solubility was less than 500 μg/mL at lower HPBCD concentrations, such as 0.1 to 3.75 mM HPBCD. This indicates that the 734THIF may not all be embedded in HPBCD. Additionally, higher HPBCD concentrations showed better water solubility improvement of 734THIF, such as 223- and 241-fold at 5 and 10 mM of HPBCD respectively, but the increase was not linear at higher HPBCD concentrations. These results indicated that 734THIF with 5 mM HPBCD is a good ratio for preparing 734THIF inclusion complex.

### 3.2. 7HPs Enhanced Solubility through Morphology and Amorphous Transformation

**Figure 2:**
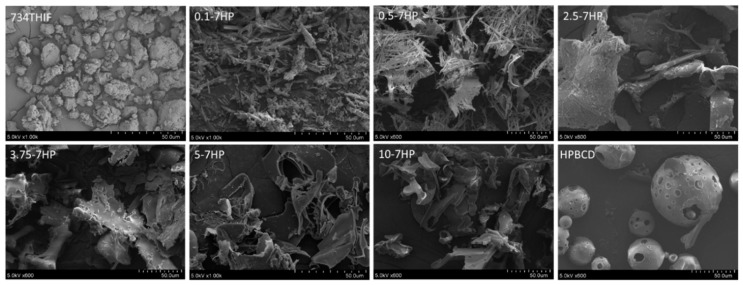
The surface morphology of 734THIF, HPBCD and 7HPs was observed by scanning electron microscopy (SEM).

The solubility increment might be due to surface area enhancement and structural amorphous transformation [18]. Surface area and morphology was observed by SEM. As seen in Figure 2, the surface morphology of 734THIF displayed uneven massive structures with non-smooth surface, while HPBCD appeared as hollow spherical particles. Inclusion complexes of 7HP with low HPBCD content, such as 0.1 to 2.5-7HP, showed irregular long rectangular structures. The long rectangular shape was gradually decreased and transformed to smooth, large flakes with large surface area when the molarity of HPBCD increased, such as 3.75-7HP, 5-7HP and 10-7HP. This indicates that 734THIF can be embedded into HPBCD and thereby enhance the water solubility of the original compound. Therefore, the shape of 734THIF and HPBCD showed drastic changes following complex formation. A similar result was found in 3-(2-isothiocyanatoethyl)-5-methoxy-1H-indole inclusion complex [18]. 

**Figure 3:**
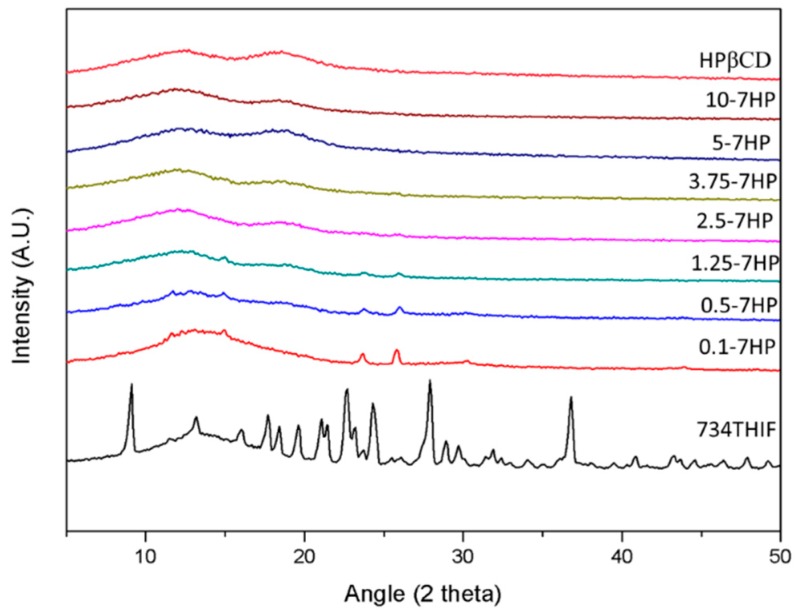
Powder X-ray diffraction patterns of HPBCD, different ratios of 734HP, and raw 734THIF.

In addition, amorphous transformation of active compound can effectively increase the water solubility of the raw compound by changing from a crystalline to an amorphous state. The XRD graphs of 734THIF, HPBCD and 7HPs are shown in Figure 3. The characteristic peaks of 734THIF appeared at diffraction angles of 9.1°, 22.7°, 24.3°, 27.9°, and 36.8°, indicating that it displayed a crystalline structure. There were several crystalline peaks displayed in 734THIF containing 0.1 to 1.25 mM HPBCD, while higher ratio formulations such as 734 THIF with 2.5 to 10 mM HPBCD, did not show crystalline peaks. The results indicate that 7HP inclusion complex formation led to amorphous transformation of 734THIF and resulted in water solubility enhancement of raw 734THIF (Table 1). Similar results have been found in quercetin [29].

### 3.3. 734THIF Interacted with Cyclodextrin by Hydrogen Bonding

**Figure 4:**
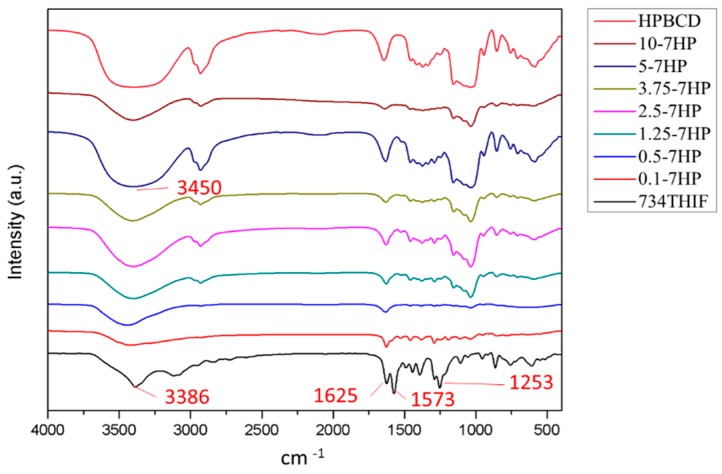
FT-IR spectra of 734THIF, HPBCD, and different ratios of 734HP.

Improvement of physicochemical properties of active compounds, such as hydrogen-bonding interaction with excipients, can effectively increase the water solubility of raw compounds after cyclodextrin inclusion complex formation. There are several characteristic peaks found in the FTIR spectra of 734THIF (Figure 4), including a broad band of phenolic–OH group at 3420–3100 cm^−1^, aromatic C=C stretching band at 1573 cm^−1^, carbonyl group (C=O) stretching vibration at 1625 cm^−1^, and C–O–H stretching band at 1253 and 1190 cm^−1^. In addition, the spectra of 7HPs showed a broader band of phenolic-OH stretch at around 3600 to 3200 cm^−1^, and lower absorption of C–O–H stretching band compared with raw 734THIF especially at high molarity (3.75 to 10-7HP) of HPBCD. These results imply that 743THIF became included into HPBCD by hydrogen bond formation with the hydrophobic group (hydroxyl propyl group).

**Figure 5:**
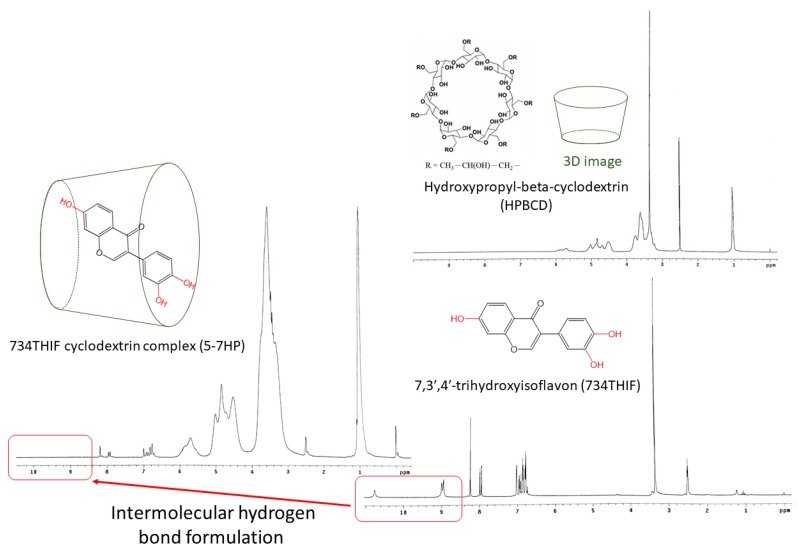
^1^H-NMR spectra and chemical structural formula of 734THIF, HPBCD, and 5-7HP.

We also used ^1^H-NMR to confirm the hydrogen bonding interactions between 734THIF and HPBCD (Figure 5). In the ^1^H-NMR spectrum of 734THIF, there are signal peaks of aromatic protons and phenolic protons in the regions of δ6.6–8.4 and δ8.9–10.5 respectively. On the other hand, the signal peaks of phenolic protons (C7, C4′ and C3′) disappeared in the ^1^H-NMR spectrum of 5-7HP. These results suggested that 734THIF was embedded into the HPBCD cavity through an intermolecular hydrogen bond formation between the phenolic–OH (C7, C4′ and C3′) group of 734THIF and the hydroxyl propyl group of HPBCD. Thus, the 734THIF inclusion complex formation with HPBCD enhanced the water solubility of 734THIF. Similar results were found by Borghetti et al., who also showed that the daidzein molecule (B and C rings) became included into the inner cavity of HPBCD and improved the solubility of raw daidzein [30].

### 3.4. Photostability of 5-7HP

**Figure 6:**
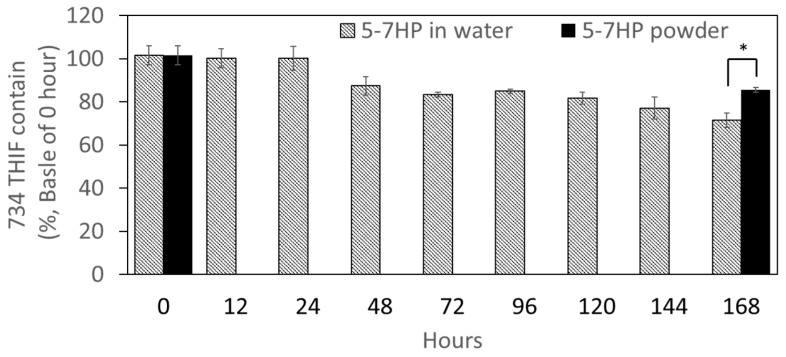
The photostability of 5-7HP powder and 5-7HP water solution. Values are mean ± SD (*n* = 4). * *p*  <  0.05.

Based on our knowledge, 734THIF is an unstable compound when exposed to light. The present study compared the photostability of 5-7HP and raw 734THIF. As shown in Figure 6, 5-7HP dissolved in water started degrading after light exposure for 48 h in a time-dependent manner. After 168 h of light exposure, 5-7HP water solution still retained 71.49 ± 3.31% of 734THIF. These results indicated that the degradation of 734THIF for all samples at various times did not exceed 30%. In addition, we also evaluated 5-7HP powder after 168 h of light exposure and its 734THIF content was 85.52 ± 1.13%. These results showed that the degradation of 5-7HP powder was lower than the 5-7HP water solution, which indicated that 5-7HP has greater photostability in dry powder form compared with the solution form. 

### 3.5. Skin Penetration Enhancement through 7HPs Formation

**Figure 7:**
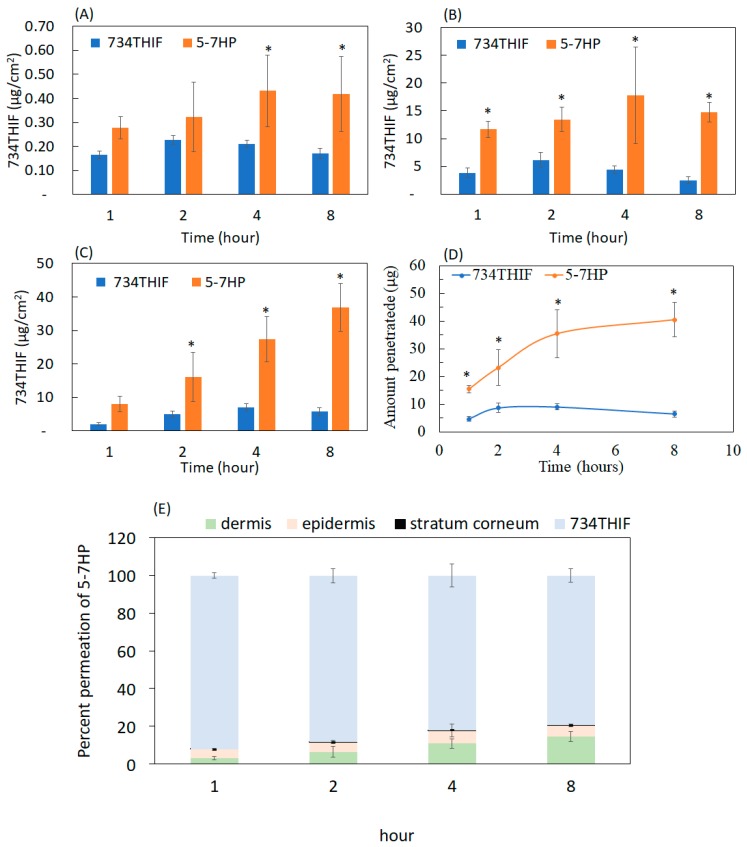
The contents of 734THIF delivered from 734THIF and 5-7HP to different skin layers: (**A**) stratum corneum, (**B**) epidermis, (**C**) and dermis. (**D**) 734THIF amount permeation in epidermis and dermis treated at different times. (**E**) Percent of 734THIF penetrated into different skin layer. Values are mean ± SD (*n* = 6). * *p*  <  0.05 compared with raw 734THIF.

Percutaneous absorption is the channeling of active ingredients through the epidermis and dermis. Poor water solubility may limit the skin penetration of active ingredients [31]. In vitro skin penetration of raw 734THIF and 5-7HP is shown in Figure 7. Following administration of raw 734THIF onto pig skin, the penetration content of 734THIF was less than 10 μg/cm^2^ in the epidermis and dermis. The highest penetration of raw 734THIF was only 9.06 ± 1.17 μg at 4 h. On the other hand, topical administration of 5-7HP led to higher penetration content of 734THIF in the epidermis and dermis compared with raw 734THIF solution (3.9-fold) in a time-dependent manner. In addition, the steady-state flux of raw 734THIF and 5-7HP was 0.048 μg/cm^2^/min and 0.19 μg/cm^2^/min respectively. In Figure 7E, the percent permeation of 5-7HP appeared to reach 20% penetration into the skin layer. These results indicate that 734THIF was effectively included into HPBCD and delivered to deeper skin layers by improving the water solubility of raw 734THIF.

### 3.6. 5-7HP Retained DPPH Scavenging Ability and Increased Cell Viability

734THIF is known as an antioxidant; however, the lipophilic properties of 734THIF might limit its pharmaceutical effects. In the present study, we evaluated the antioxidant activity of 5-7HP dissolved in H_2_O compared with 734THIF dissolved in DMSO and H_2_O through DPPH scavenging ability assay (Table 4). There was no significant difference between the scavenging 50% DPPH activity of 734D (SC_50_ = 19.49 ± 0.41 μg/mL) and 5-7HP (SC_50_ = 19.03 ± 1.03 μg/mL). In addition, 734H showed no effect on DPPH scavenging activity due to the poor solubility of 734THIF in water. Thus, 5-7HP maintained the DPPH scavenging ability of raw 734THIF and the solubility affected the antioxidant ability of 734THIF.

**Figure 8:**
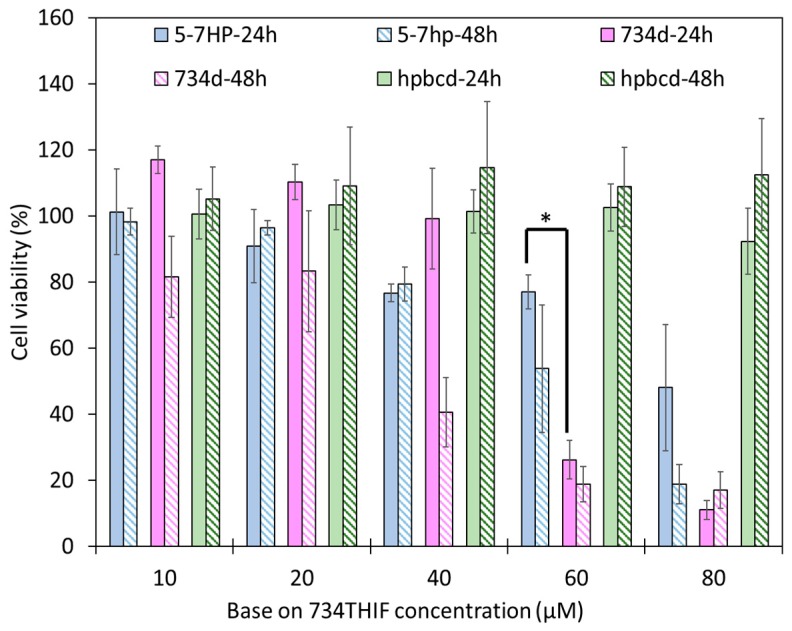
The HaCaT cell viability of 734THIF, 5-7HP and HPβCD at 24 and 48 h. All columns are mean ± SD (*n* = 3); * *p* < 0.05. HPβCD concentration was calculated by 5-7HP, which means it needs to be multiplied by 5; for example, 10 μM represent 50 μM HPβCD and so on.

A good topical formulation should possess low toxicity on cells and skin. Cell viability is one way to observe the safety of formulations and ingredients. Figure 8 shows that HPBCD is a safe excipient, since all concentrations exhibited no significant cytotoxicity. 734D at 60 μM caused 74% death of HaCaT cells at 24 h, but 5-7HP at 60 μM displayed only 20% cell death. These results indicated that 5-7HP is safer than raw 734THIF in DMSO (*p* < 0.05). In addition, 10 and 20 μM of 5-7HP resulted in 90% cell viabilities and were used for further PM-induced HaCaT cell biological study.

### 3.7. 5-7HP Inhibited PM-Induced Reactive Oxygen Species (ROS) Generation

**Figure 9:**
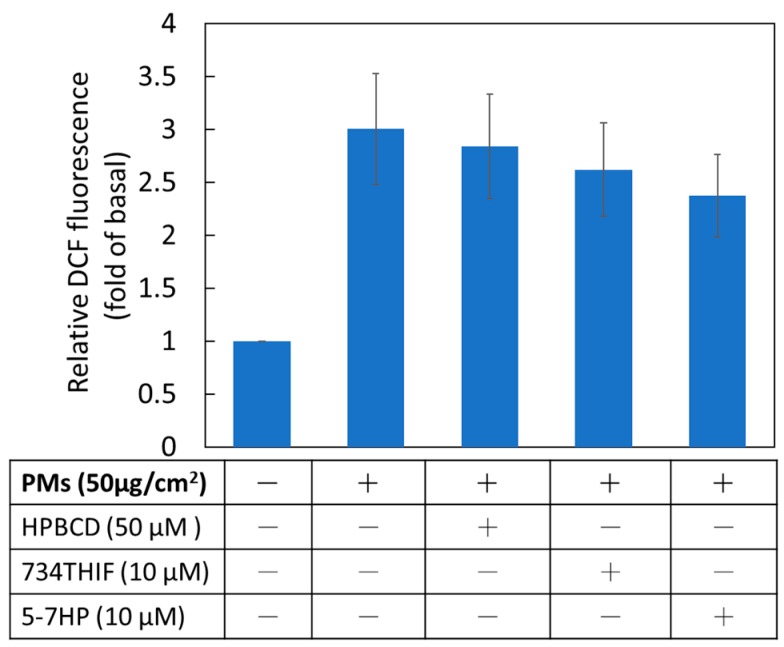
The effect of HPBCD, raw 734THIF and 5-7HP on PM-induced ROS generation in human keratinocytes. All samples (10 μM) were dissolved in DMEM solution (*n*  =  6 in each group).

ROS, a PM-induced major product, causes skin dysfunction through inducing inflammatory proteins and downregulating moisture proteins [6]. The present study indicated that PMs obviously induced ROS production 3-fold compared with non-treated PM in keratinocytes. Pretreated 5-7HP in DMEM solution downgraded PM-induced ROS generation (Figure 9). The results suggested that 5-7HP possessed antioxidant activity of 734THIF by scavenging the ROS overproduction after PM exposure.

### 3.8. 5-7HP Decreased PM-Induced Inflammation and Aging and PM-Decreased Moisture through MAPK Pathway in Keratinocytes

**Figure 10:**
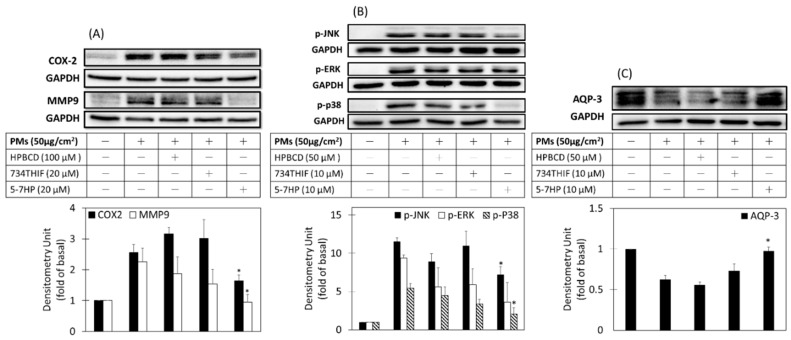
COX-2, MMP-9, AQP-3, and MAPKs protein expressions. Cells were pre-incubated with HPBCD, raw 734THIF, 5-7HP in DMEM solution for 24 h and then treated with PMs for 2 h (p-JNK, p-ERK, and p-p38, **B**); 6 h (COX-2 and MMP-9, **A**); 24 h (AQP-3, **C**). *n*  =  3 in each group; * *p*  <  0.05 compared with the group of PMs.

Lee et al. reported that PMs can increase ROS generation to disrupt the skin barrier function through activation of inflammatory pathway signaling and resulting in moisture protein downregulation in HaCaT cells [6]. Figure 10A shows that PMs increased expression of inflammatory proteins COX2 and aging protein MMP9 by 2.6- and 2.3-fold when compared to nontreated-PMs respectively. Pretreated 5-7HP significantly downregulated PM-induced COX2 and MMP9 expression but raw 734THIF did not show any inhibitory effect. The results indicated that 5-7HP can inhibit PM-induced inflammation and aging. In addition, AQP-3 expression is involved in skin hydration and is mediated by MAPKs [32]. PMs decreased AQP-3 expression and pre-treated 5-7HP significantly recovered AQP-3 levels when compared to raw 734THIF (*p* < 0.05). These results indicated that 5-7HP had moisture-retaining activity through upregulating AQP3 protein expression. 

Moreover, phosphorylation of mitogen-activated protein kinases (MAPKs), such as p-ERK, p-p38, and p-JNK, mediated the overexpression of COX2, MMP9 and AQP-3. Figure 10B shows that PMs can increase the phosphorylation of ERK, P38, and JNK when compared to control (*p* < 0.05). Pre-incubated 5-7HP reduced PM-induced p-ERK, p-p38 and p-JNK protein but raw 734THIF in DMEM solution had no significant effect on downregulating PM-induced p-ERK, p-p38, and p-JNK protein expressions. The results indicated that 5-7HP effectively decreased the expressions of inflammatory proteins (COX-2) and aging protein (MMP-9) and moisture protein (AQP-3) by downregulating the MAPKs signal pathway in the PMs-induced HaCaT cells injury model.

### 3.9. 5-7HP Enhanced Skin Hydration in Pilot Study

**Figure 11:**
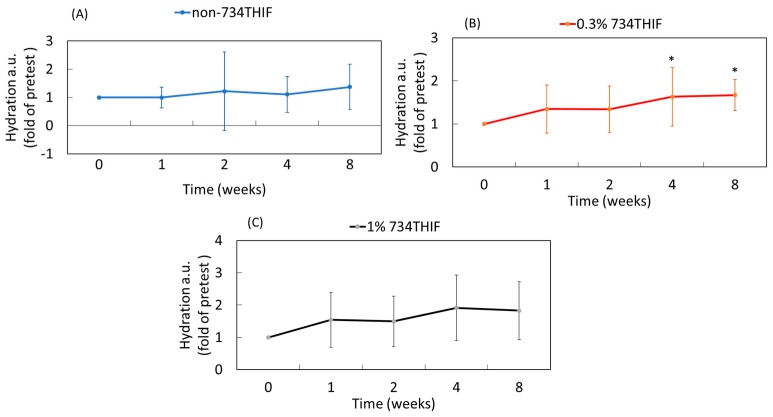
The skin surface hydration in 21 volunteers before and after treatment with different concentrations of 734THIF of 5-7HP formulation at different times. (**A**) non-content of 734THIF, (**B**) 0.3% 734THIF and (**C**) 1% 734THIF (*n*  =  7 in each group; * *p*  <  0.05 compared with the group of zero weeks).

Lee et al. revealed that PMs decreased skin hydration protein [6] and our study demonstrated that 5-7H can increase AQP-3 expression to display moisture-retaining activity following PM exposure. Therefore, we measured the moisture-retaining ability of 5-7HP formulation in a pilot study. As shown in Figure 11, the control group without 5-7HP did not display any skin hydrating effect. In addition, 5-7HP containing 0.3% 734THIF significantly increased skin surface hydration about 1.6- and 1.7-fold at 4 and 8 weeks, respectively (*p* < 0.05), when compared to before treatment use. Our data also showed that the high dose group (5-7HP containing 1% 734THIF) improved skin hydration but the result was not statistically significant. Additionally, our data also displayed high standard deviations in several groups due to individual differences between subjects. The results suggested that 5-7HP containing 0.3% 734THIF formulation provided skin hydration activity, but without any dose-dependent effect in this study. 

## 4. Discussion

According to the guideline of Biopharmaceutics Classification System (BCS) from the Food and Drug Administration (FDA), water solubility and permeability of active ingredients are major indexes that govern drug absorption and therefore are directly related to bioavailability [33]. Most compounds from products of Nature are BCS class II or IV, such as quercetin [34], resveratrol [35] and chrysin [36], and their poor water solubility may limit their absorption, resulting in reduction of biological activity. Our present study indicated that 734THIF, which belongs to BCS class II or IV, has poor water solubility, which might influence its pharmaceutical effect. Many drug delivery systems are used to improve the water solubility of lipophilic compounds, including microemulsions [37], cyclodextrin inclusion [18], nanoparticle formulations [38] and so on. HPBCD, a cyclodextrin with lipophilic inner cavities and hydrophilic outer surfaces, can interact with poorly soluble drugs to enhance drug solubility and stability [18]. HPBCD as a pharmaceutical excipient has better water solubility (600 mg/mL) than other cyclodextrins, including α-, β-, and γ-cyclodextrins (145, 18.5, and 232 mg/mL respectively) [39]. Therefore, the present study used HPBCD as an excipient to improve the low water solubility of 734THIF. The data showed that the water solubility of 734THIF was enhanced by HPBCD, and 5-7HP was the best ratio of 7HPs (Table 3 and Figure 1). Our results demonstrated that HPBCD can effectively increase the water solubility of 734THIF by improving the physicochemical properties, such as particle size reduction, hydrogen bonding formation between compound and excipient, and crystalline to amorphous transformation [18,40]. In addition, the photostability assay showed that the degradation of 734THIF did not exceed 30%. It is reasonable to propose that HPBCD can protect active compounds against environmental effects when the compound is imbedded into the cavity of HPBCD. A similar phenomenon has been shown for 3-(2-isothiocyanatoethyl)-5-methoxy-1H-indole) and lansoprazole [16,18].

The stratum corneum (SC) is the outermost layer of the skin, and acts as a skin barrier, making it hard for active compounds to pass through and exert their biological activities [41]. It is well known that skin penetration and absorption are influenced by the water solubility of raw compounds. Figure 7 indicates that raw 734THIF had poor skin penetration due to its low water solubility. Based on our best knowledge, HPBCD is a powerful chemical penetration enhancer (CPEs) used to reduce skin barrier resistance, and thus may promote the penetration of active ingredients. Our results demonstrated that 5-7HP displayed better skin penetration effect than raw 734THIF with a time-dependent effect, and the water solubility of 734 THIF was improved by inclusion into HPBCD. Furuishi et al. revealed that cyclodextrins can extract lipid and/or cholesterol from the stratum corneum and develop complexes from them, thereby decreasing skin barrier properties temporarily [42]; therefore, 5-7HP is a good pharmaceutical formulation to overcome the barrier function of the SC and subsequently increase the skin absorption of raw 734THIF. 

Active ingredients are usually unstable after pharmaceutical preparation. 734THIF is a known antioxidant and it has been revealed that 734THIF can not only suppress UV-induced skin injury through inhibition of UVB-induced COX-2 expression [12], but could improve atopic dermatitis in a mice model as well [11]. To confirm the biological activities of 734THIF after cyclodextrin inclusion complex preparation, we used a PMs-induced HaCaT keratinocyte injury model to compare their activity. It is known that PMs are associated with increased risk of skin damage, such as atopic dermatitis and skin aging [43,44]. PMs-induced skin damage may be caused by oxidative stress [45]. ROS, as an oxidative stress factor, activates MAPK proteins (phosphorylation of ERK, p38, and JNK) to mediate inflammation protein-COX2 expression and downregulate filaggrin expression [6]. In addition, phosphorylation of p38 and JNK, the major products of ROS-induced MAPK proteins, are associated with decreased expression of active AQP-3 [32]. Functions of AQP-3 include exporting glycerol into keratinocytes and associating water transporters to regulate the epidermal structure [46]; however, lack of AQP-3 decreases skin hydration, resulting in delayed wound healing and skin repair [47]. Our data showed that PMs not only induced ROS overproduction to mediate the expression of inflammation protein (COX-2) and aging protein (MMP-9), but also decreased AQP-3 expression by activating phosphorylation of JNK, ERK and p38 in HaCaT keratinocytes. Our results also demonstrated that 5-7HP dissolved in water maintains its biological activities, such as antioxidant, antiinflammation, antiaging, and moisture-retaining effects, and therefore HPBCD is a good excipient for stabilizing raw 734THIF after pharmaceutical preparation.

According to the Scientific Committee on Consumer Safety guidance, cosmetic ingredients must be evaluated for their safety [48]. In the present study, HPBCD is a safe excipient and 5-7HP appeared to have lower cell toxicity than raw 734THIF. Jiang et al. indicated that HPBCD complex formation reduces the hematological toxicity of raw 9-nitro camptothecin in an anticancer animal model when compared with a 9-nitro camptothecin-free treatment in organic solvent [49]. Moreover, our pilot study also demonstrated that there are no skin adverse effects after 5-7HP administration in human subjects, and that 5-7HP as an ingredient of topical formulation can increase the skin hydration. Skin hydration is associated with the expression of moisturizing proteins in keratinocytes, such as filaggrin and AQP-3 [50]. Furthermore, in our research, we found that 5-7HP can reverse PM-decreased AQP-3 expression. This phenomenon implies that 5-7HP increased skin hydration through AQP-3. Consequently, 5-7HP can be a safe topical moisturizing ingredient for improving human skin dryness.

## 5. Conclusions

5-7HP, 734THIF cyclodextrin inclusion complex, can successfully increase the water solubility and skin penetration of raw 734THIF. 5-7HP is not only a safe and stable cosmetic additive, but might also prevent PM-induced inflammation, aging and moisture loss in HaCaT keratinocytes. Topical lotions containing 5-7HP ingredient also showed better skin hydration effect than did raw 734THIF in a clinical study. Consequently, we suggest that 5-7HP may be an anti-pollutant additive in medicinal and cosmetic products for preventing PM-induced skin problems.

## Figures and Tables

**Table 1:** Inclusion and exclusion criteria.

Main inclusion criteria	(1) Men and women with age older than 20 years
	(2) Individuals with skin dryness
	(3) Written informed consent for participation in the trial
Main exclusion criteria	(1) Severe skin allergy, such as urticaria
	(2) Secondary infection with bacteria, fungi, or virus
	(3) Uncontrolled hypertension (SBP > 145 mmHg or DBP > 95 mmHg)
	(4) Severe liver disability (2.5-fold the normal high range value for ALT, AST)
	(5) Severe renal disability (sCr > 2.0 mg/dL)
	(6) Women who are pregnant, lactating, planning a pregnancy or women of child-bearing age who do not agree to use proper contraception
	(7) Use of oral steroids, oral antibiotic or other immunosuppressants within the past 4 weeks
	(8) Treated by systemic photochemotherapy within the past 4 weeks
	(9) History of drug abuse
	(10) Hypersensitivity to isoflavone
	(11) Use of other investigational products within the past two months
	(12) History of psychiatry disorders
	(13) History of severe immunocompromise

**Table 2:** Composition of 5-7HP lotion.

Ingredient	Negative Control	0.3% 734THIF	1% 734THIF
5-7HP	-	8.4 g	28 g
euxyl^®^ PE9010	0.05 g	0.05 g	0.05 g
Sepigel 305	2 g	2 g	2g
water	added to 100 g	added to 100 g	added to 100 g

**Table 3:** Solubility of different ratios of 7HP.

Name	HPBCD (mM)	734THIF (mM)	Solubility (μg/mL)		
	-	1	4.00	±	0.90
0.1-7HP	0.1		4.07	±	0.46
0.5-7HP	0.5		65.51	±	4.89
1.25-7HP	1.25		188.08	±	5.55
2.5-7HP	2.5		278.01	±	83.76
3.75-7HP	3.75		311.39	±	8.88
5-7HP	5		894.63	±	20.96
10-7HP	10		965.66	±	95.26

Values are mean ± SD (*n* = 3).

**Table 4:** Scavenging 50% DPPH free radicals (SC_50_) concentration of 734THIF (in water and DMSO) and 5-7HP.

Group	SC_50_ (μg/mL)
734THIF in H_2_O (734H)	NE ^a^
734THIF in DMSO (734D)	19.49 ± 0.41
5-7HP in H_2_O	19.03 ± 1.03

Values are mean ± SD (*n* = 3). ^a^ NE indicated no effect.

## References

[1] Chriscaden K., Osseiran N. WHO Releases Country Estimates on Air Pollution Exposure and Health Impact. https://www.who.int/news-room/detail/27-09-2016-who-releases-country-estimates-on-air-pollution-exposure-and-health-impact.

[2] Sun Q., Hong X., Wold L.E. (2010). Cardiovascular effects of ambient particulate air pollution exposure. Circulation.

[3] Jiménez E., Linares C., Rodríguez L.F., Díaz J. (2009). Short-term impact of particulate matter (PM2.5) on daily mortality among the over-75 age group in Madrid (In Spain). Sci. Total Environ..

[4] Sárdy M. (2009). Role of matrix metalloproteinases in skin ageing. Connect. Tissue Res..

[5] Vierkötter A., Schikowski T., Ranft U., Sugiri D., Matsui M., Krämer U., Krutmann J. (2010). Airborne particle exposure and extrinsic skin aging. J. Investig. Dermatol..

[6] Lee C.W., Lin Z.C., Hu S.C.S., Chiang Y.C., Hsu L.F., Lin Y.C., Lee I.T., Tsai M.H., Fang J.Y. (2016). Urban particulate matter down-regulates filaggrin via COX2 expression/PGE2 production leading to skin barrier dysfunction. Sci. Rep..

[7] Blaydon D.C., Kelsell D.P. (2014). Defective channels lead to an impaired skin barrier. J. Cell Sci..

[8] Young L., Je Y.J., Lee S.S., Li Z.J., Choi D.K., Kwon Y.B., Sohn K.C., Im M., Seo Y.J., Lee J.H. (2012). Changes in transepidermal water loss and skin hydration according to expression of aquaporin-3 in psoriasis. Ann. Dermatol..

[9] McDaniel D., Farris P., Valacchi G. (2018). Atmospheric skin aging-Contributors and inhibitors. J. Cosmet. Dermatol..

[10] Park J.S., Kim D.H., Lee J.K., Lee J.Y., Kim D.H., Kim H.K., Lee H.J., Kim H.C. (2010). Natural ortho-dihydroxyisoflavone derivatives from aged Korean fermented soybean paste as potent tyrosinase and melanin formation inhibitors. Bioorg. Med. Chem. Lett..

[11] Kim B.B., Kim J.R., Kim J.H., Kim Y.A., Park J.S., Yeom M.H., Joo Lee H., Lee K.W., Kang N.J. (2013). 7,3′,4′-Trihydroxyisoflavone Ameliorates the Development of Dermatophagoides farinae-Induced Atopic Dermatitis in NC/Nga Mice. Evid. Based Complement. Alternat. Med..

[12] Lee D.E., Lee K.W., Byun S., Jung S.K., Song N., Lim S.H., Heo Y.S., Kim J.E., Kang N.J., Kim B.Y. (2011). 7,3’,4’-Trihydroxyisoflavone, a metabolite of the soy isoflavone daidzein, suppresses ultraviolet B-induced skin cancer by targeting Cot and MKK4. J. Biol. Chem..

[13] Ipar V.S., Dsouza A., Devarajan P.V. (2019). Enhancing Curcumin Oral Bioavailability Through Nanoformulations. Eur. J. Drug Metab. Pharm..

[14] Tambe A., Mokashi P., Pandita N. (2019). Ex-vivo intestinal absorption study of boswellic acid, cyclodextrin complexes and poloxamer solid dispersions using everted gut sac technique. J. Pharm. Biomed. Anal..

[15] Domokos A., Balogh A., Dénes D., Nyerges G., Ződi L., Farkas B., Marosi G., Nagy Z.K. (2019). Continuous manufacturing of orally dissolving webs containing a poorly soluble drug via electrospinning. Eur. J. Pharm. Sci..

[16] Lu Y., Guo T., Qi J., Zhang J., Wu W. (2012). Enhanced dissolution and stability of lansoprazole by cyclodextrin inclusion complexation: Preparation, characterization, and molecular modeling. AAPS PharmSciTech.

[17] Loftsson T., Brewster M.E. (2012). Cyclodextrins as functional excipients: Methods to enhance complexation efficiency. Int. J. Pharm. Sci. Res..

[18] Michalska P., Wojnicz A., Ruiz-Nuño A., Abril S., Buendia I., León R. (2017). Inclusion complex of ITH12674 with 2-hydroxypropyl-β-cyclodextrin: Preparation, physical characterization and pharmacological effect. Carbohydr. Polym..

[19] Nguyen T.A., Liu B., Zhao J., Thomas D.S., Hook J.M. (2013). An investigation into the supramolecular structure, solubility, stability and antioxidant activity of rutin/cyclodextrin inclusion complex. Food Chem..

[20] Pandya P., Gattani S., Jain P., Khirwal L., Surana S. (2008). Co-solvent Evaporation Method for Enhancement of Solubility and Dissolution Rate of Poorly Aqueous Soluble Drug Simvastatin: In vitro–In vivo Evaluation. AAPS PharmSciTech.

[21] Loftsson T., Jarho P., Másson M., Järvinen T. (2005). Cyclodextrins in drug delivery. Expert Opin. Drug Deliv..

[22] Huang P.H., Tseng C.H., Lin C.Y., Lee C.W., Yen F.L. (2018). Preparation, characterizations and anti-pollutant activity of 7,3′,4′-trihydroxyisoflavone nanoparticles in particulate matter-induced HaCaT keratinocytes. Int. J. Nanomed..

[23] Andonova V., Peneva P., Georgiev G.S., Toncheva V.T., Apostolova E., Peychev Z., Dimitrova S., Katsarova M., Petrova N., Kassarova M. (2017). Ketoprofen-loaded polymer carriers in bigel formulation: An approach to enhancing drug photostability in topical application forms. Int. J. Nanomed..

[24] COLIPA Guidelines: Guidlines for Percutaneous Absorption/Penetration. http://www.jacvam.jp/files/doc/05_01/05_01_Z3.pdf.

[25] Kassis V., Søndergaard J. (1982). Heat-separation of normal human skin for epidermal and dermal prostaglandin analysis. Arch. Dermatol. Res..

[26] Huang P.H., Hu S.C.S., Lee C.W., Yeh A.C., Tseng C.H., Yen F.L. (2016). Design of acid-responsive polymeric nanoparticles for 7,3′,4′-trihydroxyisoflavone topical administration. Int. J. Nanomed..

[27] Cheon C., Park S., Park J.S., Oh S.M., Jang S., Go H.Y., Jang B.H., Shin Y.C., Ko S.G. (2013). KM110329 in adult patients with atopic dermatitis: A randomised, double-blind, placebo-controlled, multicentre trial–study protocol. BMC Complement. Altern. Med..

[28] Dittert L.W., Higuchi T., Reese D.R. (1964). Phase solubility technique in studying the formation of complex salts of triamterene. J. Pharm. Sci..

[29] Güleç K., Demirel M. (2016). Characterization and Antioxidant Activity of Quercetin/Methyl-β-Cyclodextrin Complexes. Curr. Drug Deliv..

[30] Borghetti G.S., Pinto A.P., Lula I.S., Sinisterra R.D., Teixeira H.F., Bassani V.L. (2011). Daidzein/cyclodextrin/hydrophilic polymer ternary systems. Drug Dev. Ind. Pharm..

[31] Yotsumoto K., Ishii K., Kokubo M., Yasuoka S. (2018). Improvement of the skin penetration of hydrophobic drugs by polymeric micelles. Int. J. Pharm..

[32] Huang L.Z., Li F.X., Dong C., Wang J.P., Wu H.J., Shuang S.M. (2015). Down-regulation of aquaporin3 expression by lipopolysaccharide via p38/c-Jun N-terminal kinase signalling pathway in HT-29 human colon epithelial cells. World J. Gastroenterol..

[33] Waiver of In Vivo Bioavailability and Bioequivalence Studies for Immediate-Release Solid Oral Dosage Forms Based on a Biopharmaceutics Classification System Guidance for Industry. https://www.fda.gov/regulatory-information/search-fda-guidance-documents/waiver-vivo-bioavailability-and-bioequivalence-studies-immediate-release-solid-oral-dosage-forms.

[34] Seo J.Y., Pandey R.P., Lee J., Sohng J.K., Namkung W., Park Y.I. (2019). Quercetin 3-O-xyloside ameliorates acute pancreatitis in vitro via the reduction of ER stress and enhancement of apoptosis. Phytomedicine.

[35] Salem H.F., Kharshoum R.M., Abou-Taleb H.A., Naguib D.M. (2019). Nanosized Transferosome-Based Intranasal In Situ Gel for Brain Targeting of Resveratrol: Formulation, Optimization, In Vitro Evaluation, and In Vivo Pharmacokinetic Study. AAPS PharmSciTech.

[36] Baidya D., Kushwaha J., Mahadik K., Patil S. (2019). Chrysin-loaded folate conjugated PF127-F68 mixed micelles with enhanced oral bioavailability and anticancer activity against human breast cancer cells. Drug Dev. Ind. Pharm..

[37] Sieniawska E., Œwi¹tek £., Wota M., Rajtar B., Polz-Dacewicz M. (2019). Microemulsions of essentials oils–Increase of solubility and antioxidant activity or cytotoxicity?. Food Chem. Toxicol..

[38] El-Laithy H.M., Badawi A., Abdelmalak N.S., Elsayyad N.M.E. (2019). Stabilizing excipients for engineered clopidogrel bisulfate procubosome derived in situ cubosomes for enhanced intestinal dissolution: Stability and bioavailability considerations. Eur. J. Pharm. Sci..

[39] Challa R., Ahuja A., Ali J., Khar R.K. (2005). Cyclodextrins in drug delivery: An updated review. AAPS PharmSciTech.

[40] Zhang W., Gong X., Cai Y., Zhang C., Yu X., Fan J., Diao G. (2013). Investigation of water-soluble inclusion complex of hypericin with â-cyclodextrin polymer. Carbohydr. Polym..

[41] Lane M.E. (2013). Skin penetration enhancers. Int. J. Pharm..

[42] Furuishi T., Takahashi S., Ogawa N., Gunji M., Nagase H., Suzuki T., Endo T., Ueda H., Yonemochi E., Tomono K. (2017). Enhanced dissolution and skin permeation profiles of epalrestat with â-cyclodextrin derivatives using a cogrinding method. Eur. J. Pharm. Sci..

[43] Kim Y.M., Kim J., Jung K., Eo S., Ahn K. (2018). The effects of particulate matter on atopic dermatitis symptoms are influenced by weather type: Application of spatial synoptic classification (SSC). Int. J. Hyg. Environ. Health.

[44] Park S.Y., Byun E.J., Lee J.D., Kim S., Kim H.S. (2018). Air Pollution, Autophagy, and Skin Aging: Impact of Particulate Matter (PM10) on Human Dermal Fibroblasts. Int. J. Mol. Med..

[45] Piao M.J., Ahn M.J., Kang K.A., Ryu Y.S., Hyun Y.J., Shilnikova K., Zhen A.X., Jeong J.W., Choi Y.H., Kang H.K. (2018). Particulate matter 2.5 damages skin cells by inducing oxidative stress, subcellular organelle dysfunction, and apoptosis. Arch. Toxicol..

[46] Sougrat R., Morand M., Gondran C., Barré P., Gobin R., Bonté F., Dumas M., Verbavatz J.M. (2002). Functional expression of AQP3 in human skin epidermis and reconstructed epidermis. J. Investig. Dermatol..

[47] Hara M., Ma T., Verkman A.S. (2002). Selectively reduced glycerol in skin of aquaporin-3-deficient mice may account for impaired skin hydration, elasticity, and barrier recovery. J. Biol. Chem..

[48] The SCCS Notes of Guidance for the Testing of Cosmetic Ingredients and Their Safety Evaluation 10th Revision. https://ec.europa.eu/health/sites/health/files/scientific_committees/consumer_safety/docs/sccs_o_224.pdf.

[49] Jiang Y., Jiang X., Law K., Chen Y., Gu J., Zhang W., Xin H., Sha X., Fang X. (2011). Enhanced anti-tumor effect of 9-nitro-camptothecin complexed by hydroxypropyl-â-cyclodextrin and safety evaluation. Int. J. Pharm..

[50] Verdier-Sévrain S., Bonté F. (2007). Skin hydration: A review on its molecular mechanisms. J. Cosmet. Dermatol..

